# Quality Traits and Nutritional Value of Pork and Poultry Meat from Animals Fed with Seaweeds

**DOI:** 10.3390/foods10122961

**Published:** 2021-12-01

**Authors:** David Miguel Ribeiro, Cátia Falcão Martins, Mónica Costa, Diogo Coelho, José Pestana, Cristina Alfaia, Madalena Lordelo, André Martinho de Almeida, João Pedro Bengala Freire, José António Mestre Prates

**Affiliations:** 1 LEAF—Linking Landscape, Environment, Agriculture and Food, Instituto Superior de Agronomia, Universidade de Lisboa, Tapada da Ajuda, 1349-017 Lisboa, Portugal; davidribeiro@isa.ulisboa.pt (D.M.R.); catiamartins@isa.ulisboa.pt (C.F.M.); mlordelo@isa.ulisboa.pt (M.L.); aalmeida@isa.ulisboa.pt (A.M.d.A.); jpfreire@isa.ulisboa.pt (J.P.B.F.); 2 CIISA—Centro de Investigação Interdisciplinar em Sanidade Animal, Faculdade de Medicina Veterinária, Universidade de Lisboa, Avenida da Universidade Técnica, 1300-477 Lisboa, Portugal; monicacosta@fmv.ulisboa.pt (M.C.); diogocoelho@fmv.ulisboa.pt (D.C.); jpestana@fmv.ulisboa.pt (J.P.); cpmateus@fmv.ulisboa.pt (C.A.)

**Keywords:** meat quality, nutritional value, pork, poultry, seaweeds

## Abstract

Seaweeds have caught the attention of the scientific community in recent years. Their production can mitigate the negative impact of anthropogenic activity and their use in animal nutrition reduces the dependency on conventional crops such as maize and soybean meal. In the context of monogastric animals, novel approaches have made it possible to optimise their use in feed, namely polysaccharide extraction, biomass fermentation, enzymatic processing, and feed supplementation with carbohydrate-active enzymes (CAZymes). Their bioactive properties make them putative candidates as feed ingredients that enhance meat quality traits, such as lipid oxidation, shelf-life, and meat colour. Indeed, they are excellent sources of essential amino acids, polyunsaturated fatty acids, minerals, and pigments that can be transferred to the meat of monogastric animals. However, their nutritional composition is highly variable, depending on species, harvesting region, local pollution, and harvesting season, among other factors. In this review, we assess the current use and challenges of using seaweeds in pig and poultry diets, envisaging to improve meat quality and its nutritional value.

## 1. Introduction

The worldwide human population is expected to increase to over 9 billion by 2050 [1], predicting to double the current demand for meat products [2]. Pork and poultry meat are the two most consumed meats in the world [3]. Their production is largely dependent on intensive systems that use maize and soybean meal as major dietary sources of energy and crude protein, respectively. The European feed industry is largely dependent on the imports of these feedstuffs from other countries, namely Brazil, Argentina, and the USA. The production of these feedstuffs requires large land areas and high resource input, including water and pesticides. Their production has been reported as being a contributor to deforestation in South America [4]. Therefore, it is of paramount importance to find alternatives to these crops that are economically and environmentally viable in the long-term, mitigating the feed–food–fuel competition. In recent years, the scientific community has dedicated its attention to finding such alternatives, from insects [5,6] to food industry by-products [7,8] and marine resources, including microalgae [9,10] and macroalgae [11,12].

Macroalgae, or seaweeds, are multicellular, fast-growing algae classified into three main groups: *Phaeophyceae* (brown algae), *Rhodophyceae* (red algae), and *Chlorophyceae* (green algae) [2]. Seaweeds have a plethora of applications and have been used for several centuries. For example, harvesting *moliço* (the combined biomass of *Zostera marina*, *Zostera noltei*, and *Ulva* sp., among other species) to use as fertiliser in small-scale agriculture was a major economic activity in the Ria de Aveiro region in Northern Portugal during the 18th century [13]. Within the context of animal nutrition, using seaweeds as a feedstuff also has a historical background. For instance, in the Scottish Island of North Ronaldsay, in the Orkney archipelago, sheep have grazed on a nearly exclusively seaweed diet since the 19th century [14]. In the 20th century, farm animals in Iceland were fed with dried seaweeds, typically during winter [15]. During the Second World War, they were used as a feedstuff in Europe due to the scarcity of other nutrient sources [16]. Following this, there was a hiatus in their interest due to the availability of higher quality feedstuffs, such as maize or soybean meal, and to the fact that there was no technology that dealt with their anti-nutritional factors, particularly for monogastric animals. In addition, the cultivation of macroalgae can be associated with environmental and economic issues [2]. Seaweeds were given renewed attention in recent years, mostly due to their potential to reduce the environmental impact of production systems, such as mitigating eutrophication and carbon emissions. Indeed, using seaweed as a feedstuff was made possible by using sustainable algae production systems, such as integrated multi-trophic aquaculture (IMTA), and recent technology that allows the efficient processing of such rich biomass [2]. However, fresh seaweed biomass is bulky, heterogeneous, and prone to spoilage. Several methods, such as fermentation and drying followed by milling, increase storage periods and nutritional homogeneity and allow their incorporation into animal diets [17]. Moreover, technologies aimed at tackling anti-nutritional properties related to their recalcitrant cell wall (e.g., fermentation and CAZyme supplementation) are extremely relevant when considering the dietary incorporation of seaweeds in monogastric animal diets [2,18]. In recent years, the chemical characterisation of seaweed extracts has found them to be immensely versatile. Laminarin, for example, a storage polysaccharide from brown seaweeds, has anti-tumoral activity [19], cosmetic applications in skin care [20], and prebiotic properties [21]. As their use in animal nutrition is becoming increasingly reported, it is important to evaluate the impact of dietary seaweeds, including their derived products, on meat quality traits.

Pork and poultry meat are good sources of protein, minerals, vitamins, and other bioactive compounds and are easily accessible to modern consumers, with a presence in most cultures worldwide. Meat quality is a major concern for the industry because it is a determinant factor in consumer acceptability, which has increasing demands for healthy and nutritious products. Water holding capacity (WHC), tenderness, colour, lipid oxidation, flavour, and shelf-life are among the factors that determine meat quality [22]. In addition, the nutrient profile of meat is also important in this evaluation, particularly regarding fatty acid (FA), amino acid, and mineral profiles. Controlling these quality parameters is of paramount importance since changing one is enough to sway a number of other inter-related factors. Pork, for example, is dependent on its intramuscular fat (IMF) content, ideally above 2.5%, to have desirable organoleptic properties. However, the genetic selection of modern breeds for reduced subcutaneous fat has a detrimental effect on the IMF content, and consequently, on meat quality [23]. Concomitantly, it is important to consider the FA profile of meat due to its relation to metabolic disorders in humans [24]. Indeed, fast-growing breeds have high proportions of saturated fatty acids (SFA) in their meat, including lauric (C12:0), myristic (C14:0), and palmitic (C16:0) acids, whose high intake may contribute towards occlusive arterial lesions [25,26]. To contradict this effect, increasing n-3 polyunsaturated fatty acid (PUFA) and reducing SFA content is feasible. However, increasing PUFA might increase the oxidative potential of meat. Concerning poultry, particularly broiler chickens, the genetic selection for increased growth rates, heavier carcasses, and high breast meat yields has increased the incidence of abnormalities, such as deep pectoral myopathy and white striping. These not only worsen meat visual aspects, but also contribute to the development of pale, soft, and exudative (PSE) meat, characterised by low WHC and shelf-life [27]. Therefore, dietary approaches are a viable option to mitigate these effects [28]. For instance, feeding 10% *Chlorella vulgaris* to broiler chickens has improved meat yellowness (b*) through the accumulation of dietary carotenoids in breast and thigh meat [29]. The same happened when broiler chickens were fed with 15% *Spirulina platensis*, albeit while increasing SFA and decreasing n-3 PUFA in breast and thigh meats [30], which has a negative effect on its nutritional value. Therefore, when aiming to improve meat quality, all factors should be considered given the trickle-down effects that may occur.

**Figure 1:**
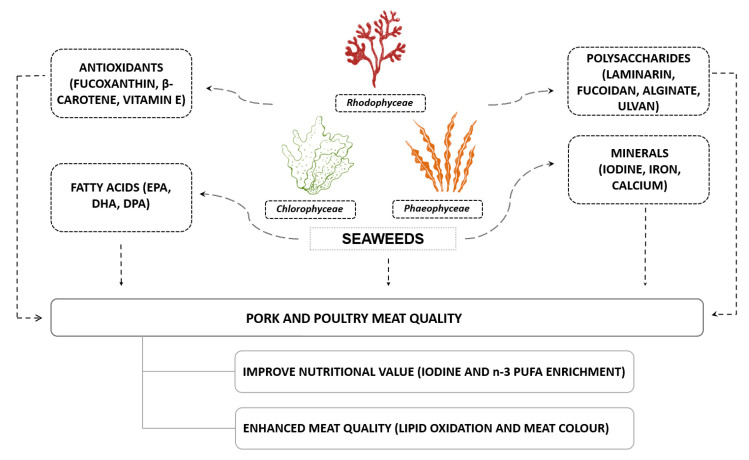
Potential benefits of seaweed-containing diets on quality and nutritional value of pork and poultry meat.

Some meat quality parameters have a genetic predisposition [31,32] but most can be manipulated by different dietary approaches. Indeed, pigment-rich sources, such as microalgae [33], and unsaturated fatty acid sources, such as linseed [34], have been used to manipulate meat colour and fatty acid profile, respectively. The potential effects of seaweed inclusion on meat quality are depicted in Figure 1. The use of seaweeds in animal nutrition has been reviewed in the last decade [2,16,35,36]. However, one systematic review exclusively dedicated to the effect of dietary seaweeds on monogastric meat quality and its nutritional value is, to our knowledge, unavailable. Therefore, the objective of this review was to analyse the currently available literature that has reported the effects of using dietary seaweeds, or their derived products, on meat quality and related nutritional value of monogastric animals, with an emphasis on pork and chicken, the two most consumed meats worldwide.

## 2. Seaweed Production—A Brief Overview

In order to contextualise how seaweeds can be produced for the feed industry, we provide a brief overview on its state of the art. According to the FAO, worldwide annual production of seaweed biomass reached 34,554,366 metric tonnes in 2019, with Asia being the major contributor [37]. China alone contributes 58% of world production, while Europe’s production is around 3.2%. The most produced genera are *Sacharina*, *Undaria*, *Porphyra*, *Euchema,* and *Gracilaria*, representing 98% of produced seaweed [38]. Seaweed production can include the harvesting of naturally available biomass or its production in controlled conditions. In the former modality, seaweeds can be manually or mechanically harvested from the shoreline before being sorted and further processed. This has been reported in countries such as Canada that are taking advantage of the beach-cast biomass of species such as *Mazzaella japonica* [39]. The latter can be performed onshore or offshore, producing only seaweeds or together with other organisms in IMTA systems [40]. Offshore cultivation is performed in confined spaces (tanks, ponds, lagoons) and allows quality monitoring and manipulation of production conditions (light, nutrient concentration, pH, O_2_, CO_2_). However, it has higher production costs compared to onshore cultivation due to its dependence on infrastructure and high maintenance costs. Onshore cultivation is the cheapest alternative and has the advantage of not requiring arable land area. It allows for manipulating the costs by choosing different substrates (seabed, lines, nets) and cultivation (seedlings or transplantation) methods. It is more susceptible to environmental conditions and pests and has lower nutrient availability [41]. This is where IMTA systems are useful, taking advantage of organisms in different trophic levels to increase nutrient circulation [40]. Producing fish (e.g., *Sciaenops ocellatus*) in combination with seaweeds (e.g., *Porphyra dioica*, *Porphyra umbilicalis*, *Gracilaria vermiculophyla*, *Ulva rigida*) and/or bottom feeders (e.g., *Holothoria scabra*, caprellids) enables the utilisation of organic waste from uneaten feed, faeces, and deteriorating kelp blades [42,43,44,45,46]. Finally, seaweed aquaculture has several positive externalities, including the maintenance of local populations, the creation of small ecosystems, and water quality enhancement through the assimilation of phosphorous and nitrogen, allowing the mitigation of eutrophication events [47,48,49].

The methods employed for processing seaweeds depend on their final utilisation. For feed and pharmaceutical applications, they can be dried prior to feed manufacturing or compound extraction, respectively. For human consumption, drying may not be necessary at all. The freshly caught biomass is first washed to remove excess salt and other unwanted elements. Due to its perishable nature and high moisture content (around 80%), it is often necessary to dry [50]. This increases the shelf-life and reduces its volume significantly. These are particularly important aspects for long-term storage. They can be dried by direct sunlight or with mechanical dryers (e.g., oven or freeze dryers). Choosing the drying process works to condition the nutritional composition of seaweeds [50]. For example, it has been reported that freeze drying *Sargassum hemiphyllum* preserves amino acids, total PUFA, and vitamin C to a greater extent compared to sun and oven drying [51]. Neoh et al. [52] have reported that vacuum drying *Kappaphycus alvarezii* preserves its total phenolic content while its sundried biomass has the lowest antioxidant activity compared to vacuum, freeze, and oven drying. If one wants to extract bioactive compounds, there are several combinations of drying and extraction techniques appropriate for each target, as summarised by Kadam et al. [50]. For it to be used in monogastric animal diets, the dried biomass is milled in order to be included in compound feeds in the form of a meal. The steps prior to animal consumption are very important because they can affect the nutritional profile of seaweeds and, therefore, its effects on animal product quality. The nutritional properties of seaweed meals are reviewed in the following section.

## 3. Nutritional Properties of Seaweeds

Seaweeds have a very heterogeneous nutritional composition. They vary depending on the species, harvest season, harvesting site, *post*-harvest processing, among other factors [2]. A recent review on the nutritional composition of several seaweed species is presented in Table 1. This subject has been extensively reviewed in recent years. For further details about this aspect, we refer to another publication by our team [2].

Brown seaweeds (*Phaeophyceae*) attribute their colour to the highest presence of a brown pigment—fucoxanthin—in relation to others, including chlorophylls a and c, and β-carotene [16]. They have a low crude fat content, ranging from 0.5 to 6.5% on a dry matter (DM) basis when considering *Ascophylum* sp., *Laminaria* sp., and *Undaria pinnatifida* [2]. Compared to the other two groups, brown seaweeds also have a low crude protein (CP) content. For instance, the CP levels of *Sacharina latissima* and *Ascophylum nodosum* were reported to range between 11 and 16% [11], whereas *Laminaria japonica* has 21% CP [53]. They have high contents of crude fibre with different polysaccharides, mainly alginate, fucoidan, and the storage polysaccharide (β-1,3 glucan) laminarin [56]. As these polysaccharides are resistant to hydrolysis in the upper digestive tract, their use has been considered in the prebiotic form [57]. Brown seaweeds also have high crude ash contents that can reach up to 35% in DM [16]. Among its mineral composition, iodine (I) is particularly high given that these seaweeds can easily assimilate it from seawater. The colour of green seaweeds (*Chlorophyta*) is due to the higher proportion of chlorophylls compared to β-carotene and other xanthophylls. Their protein content is higher than in brown seaweeds and lower than in red seaweeds. The CP content of *Ulva* sp., for example, can reach 42% of DM. Their carbohydrate content is the highest among taxonomic groups [2], having ulvan as the main cell wall polysaccharide. Their main reserve polysaccharide is starch [21]. Red seaweeds (*Rhodophyceae*) are red due to the presence of two biloprotein pigments: R-phycoerythrin and R-phycocyanin. Several species, such as *Porphyra*, have CP contents similar to that of soybean meal, up to 50% in DM [16]. Their main reserve polysaccharide is floridian starch, similar to land-plant starch, without amylose. Red seaweeds are also an abundant source of carrageenan and agar [21].

Nevertheless, seaweeds can also have anti-nutritional factors and other components that are particularly important to be aware of in the context of monogastric nutrition. Indeed, they may accumulate pollutants and heavy metals such as arsenic (As). Arsenic is mostly present as arsenosugars, which are not toxic, but As accumulation in the environment is possible through manure [17]. Brown seaweeds, such as *A. nodosum* and *Fucus serratus*, have phlorotannins responsible for lower in vitro digestibility of pig feed [58]. Moreover, seaweed cell wall polysaccharides can compromise feed digestibility depending on their various degrees of complexity, i.e., the degree of polymerisation and the number of cross-chain links. Lastly, there is the case of *post*-harvesting treatment, which can affect composition by degrading desirable pigments in the case of air drying. The fact that seaweeds may compromise feed digestibility explains why most literature concerning the use of seaweeds in animal nutrition reports only very low levels of incorporation, focusing instead on their promising prebiotic effects.

## 4. Effect of Dietary Seaweeds on Pork Meat Quality

The dietary inclusion of seaweeds in pig feed has been reported in two different ways, using the whole biomass or as polysaccharide extracts. Feeding pigs with the whole biomass poses major challenges, mostly due to the anti-nutritional effect of their complex polysaccharides and phlorotannins. The levels of dietary incorporation of seaweed biomass are generally found to be below 4%, which are considered as additive/supplement levels. This is most likely due to the low digestibility of seaweed polysaccharides in pigs. The development of strategies that allow the use of these seaweeds as ingredients are undoubtedly necessary. Moreover, extracting the biomass is the most efficient way to take advantage of various bioactive molecules, such as laminarin, fucoidan, and alginate, which have prebiotic properties. This section describes the effect of feeding either the whole biomass or extracts on the meat quality traits of pigs. The different results found are summarised in Table 2.

The use of macroalgae as whole biomass was shown to modify meat colour and mineral composition. Jerez-Timaure et al. [59] have reported that feeding fattening pigs with up to 4% *Macrocystis pyrifera* influenced meat colour, with the 4% group having less red intensity (a*) compared to either the control or the group fed 2% seaweed. The former also had less iron (Fe) in its meat. Iron is a major constituent of myoglobin, a haemoprotein which is a determinant factor for meat colour [64]. The colour of meat develops with the oxidation of deoxymyoglobin into oxymyoglobin and metmyoglobin, developing the colour from reddish to brownish [65]. It has been reported that the presence of antioxidants such as vitamin E prevents myoglobin oxidation [66]. Hence, the lower red intensity of meat from pigs fed with 4% *Macrocystis pyrifera* could be related to the highest availability of antioxidants from the seaweed that prevent myoglobin oxidation and therefore, colour development. In addition, the lowest red intensity could result from the lowest number of muscle oxidative fibres [67]. Moreover, the lowest availability of dietary Fe could contribute to these differences. Interestingly, the group that was fed with more seaweed also had less manganese (Mn) and copper (Cu) in its meat compared to the control animals. The lower digestive availability of these three microminerals is putatively caused by the formation of insoluble complexes with other feed components. Michalak et al. [63] enriched *Enteromorpha* sp. biomass with Cu and zinc (Zn) and included it in the diet of fattening pigs. They found that seaweed inclusion lowered crude ash digestibility by 15% when compared to the controls without having statistically significant effects on either meat quality or carcass characteristics. However, this negative effect on ash/mineral digestibility does not seem to prevent iodine accumulation, which has been reported in pigs supplemented with 2% *A. nodosum* [60].

Seaweed polysaccharides such as laminarin, fucoidan, and ulvan have antibiotic and antioxidative properties [21]. Fucoidan, for example, has higher antioxidant capacity compared to laminarin due to the higher degree of sulphate groups and positive charge [68]. As such, they have been used as an alternative to antibiotics as growth promotors [69,70] and in meat-derived product formulations to enhance shelf-life [71]. Moroney et al. [61] have demonstrated that the dietary inclusion of polysaccharides from *Laminaria digitate*—laminarin (500 mg/kg) and fucoidan (420 mg/kg)—decreased lipid oxidation in the *longissimus dorsi* muscle of pigs. These authors later found that this was also achieved with 450 and 900 mg/kg of laminarin/fucoidan extract. Similar results were obtained by Rajauria et al. [72] where 180 mg/kg laminarin and 330 mg/kg fucoidan supplementation improved the total antioxidant capacity of *longissimus dorsi* steaks packed in modified atmosphere for 14 days, albeit reducing redness (a*) after 4 days of storage. It has been demonstrated that both of these polysaccharides are absorbed during digestion [68]. Their consequent presence in tissues could increase their oxidative stability by neutralising ROS [73], however, studies have reported that dietary supplementation with laminarin and fucoidan does not improve free radical scavenging activity in pork [62], suggesting that they are transformed post-absorption. Hence, the positive effects on lipid oxidation likely derive from other sources. Firstly, these polysaccharides may positively influence the gut environment, thus improving overall immune function i.e., reactive-oxygen species (ROS) scavenging activities. Indeed, the dietary supplementation of piglet diets with 300 mg/kg laminarin has been reported to reduce the abundance of gut *Enterobacteriaceae*, which contributes to post-weaning stress [74]. Secondly, they also influence the fatty acid profile of meat, whose composition is of major influence for meat oxidation. This profile is also partly influenced by the microbiome since the short-chain fatty acids that are therein absorbed are substrates for endogenous synthesis of other fatty acids. Indeed, Moroney et al. [62] found that laminarin/fucoidan supplementation decreases the total SFA in meat by lowering the levels of stearic (C18:0) and arachidic (C20:0) acids in the *longissimus dorsi* of pigs. To sum up, dietary seaweed extracts can indirectly influence the lipid oxidation of meat, mediated by modulating the gut microbiome of pigs.

## 5. Effect of Dietary Seaweeds on Poultry Meat Quality

Similar to pigs, poultry have been fed with seaweeds either in an intact biomass form or with polysaccharide extracts [75], whose dietary inclusion rates are generally low. The main effects of seaweed inclusion on poultry meat quality traits are summarised in Table 3.

Ahmed et al. [76] reported that feeding *L. japonica* (0.5%) fermented with *Bacillus subtilis* and *Aspergillus oryzae* improved chicken meat oxidative stability. Matshogo et al. [82] treated *Ulva* sp. with different rates of fibrolytic enzymes (cellulase, hemicellulase, arabinase, β-glucanase, and xylanase) before feeding it to Cobb 500 broilers. They found a linear increase in hot carcass weight in response to increasing enzymatic treatment rates and a linear decrease in breast lightness (L*). The reduced lightness of breast meat might originate from the increasing availability of intracellular pigments as a result of enzymatic disruption of cell wall polysaccharides. This has been reported previously in Ross 308 broilers fed with Spirulina, which accumulated more total carotenoids [30]. The effect of dietary seaweed on meat colour has also been reported in Broad Breasted Bronze turkeys fed 1% and 2% *A. nodosum*, where redness intensity (a*) was increased in both breast and thigh meat in response to dietary treatments [78]. The latter seaweed has also been fed to broilers with up to 2% level of inclusion without significant effects on lipid oxidation of breast meat. However, the dietary treatments with 1% and 2% significantly increased γ-linolenic acid (C18:3 n-6) compared to the control [79]. The dietary incorporation of 3% *Ulva lactuca* (replacing maize) has been reported to increase dressing and breast yield while reducing the abdominal fat percentage of broiler chickens [81]. Supplementing broiler chicken diets with polymannuronate, an alginate-derived compound extracted from brown macroalgae, has resulted in reduced lipid oxidation. This also led to an increased glutathione peroxidase, albeit only in the two groups with the lowest supplementation (0.1 and 0.2%), which corroborates the ability of the algal extracted polysaccharides to act as antioxidants [83].

Other studies have described an absence of effects on Vencobb 400 chicken meat quality by feeding *Kappaphycus alvarezii* extract [80]. The lack of effects could be a consequence of the low levels of dietary incorporation of up to 0.5%. Islam et al. [77] have supplemented duck diets with a mixture of *L. japonica* and charcoal to act as a growth promotor, replacing dietary antibiotics. Their incorporation levels were lower than 1%, and yet they achieved cholesterol reduction in meat, as well as reduced lipid oxidation compared to control. The mechanism by which this is achieved is uncertain, but could derive from improved gut health, similarly to what has been described before in pigs. Therefore, the responses of different poultry and algae species are factors to consider while using seaweeds as supplements, particularly given the heterogenous nature of the chemical composition of seaweeds.

## 6. Future Challenges

The number of publications available on the use of dietary seaweeds in poultry and pig diets aiming to improve meat quality traits is incipient compared to other alternative feed sources [2]. With the advent of novel technologies, we expect that the interest in such feedstuffs, and consequently the number of publications, will increase in coming years. It is noteworthy, however, to mention that the widespread use of this rich and abundant source of biomass is currently hindered by their high costs and the presence of different anti-nutritional factors and the consequent negative effects on the monogastric digestive system. Nonetheless, there are clear benefits of using these sources, as mentioned throughout this review. Indeed, seaweeds can potentially reduce meat oxidation and improve the shelf-life of meat products. The pigments from seaweeds can also be accumulated in animal muscle, improving meat colour. Iodine can be transferred to animal tissues, which could contribute to mitigate iodine deficiency in humans, with health benefits in thyroid disfunctions [84] and during pregnancy [85]. Moreover, feeding pigs with seaweeds enables the use of eutrophication/algal bloom biomass [63], and in the case of poultry, it can potentially contribute to reduce ammoniac emissions [76]. Macroalgae cultivation for feed applications can also emerge as a response to environmental concerns, such as the greenhouse effect, with their ability to fix carbon. Thus, seaweeds can be used to replace other conventional feedstuffs while providing bioactive substances that contribute to increased meat quality and gut health. Regardless of all this, using seaweeds as macronutrient sources poses a major challenge. *Post*-harvesting treatments (e.g., fermentation, mechanical processes) and enzymatic feed supplementation are suggested as two possible paths in order to break down the non-starch polysaccharides that hinder/limit the activity of the endogenous enzymes during the digestive process. Therefore, the anti-nutritional factors inherent to seaweeds should be considered in order to maximise the nutritional value of this abundant biomass and take advantage of its meat quality-enhancing properties.

## 7. Conclusions

Using seaweeds to enhance the meat quality of monogastric animals, particularly poultry and pigs, shows great potential. However, the aforementioned digestive implications need to be considered in future research to maximise the benefits drawn from this novel feedstuff.

## Figures and Tables

**Table 1:** Nutritional composition of dried meals from brown, green, and red seaweeds. Units in percentage on a dry matter basis. NDF—neutral detergent fibre, ADF—acid detergent fibre, ADL—acid detergent lignin.

Seaweed	Dry Matter	Ash	Crude Protein	Crude Fat	Crude Fibre	NDF	ADF	ADL	Reference
*Phaeophyceae* (brown)									
*Laminaria japonica*	97.5	14.9	20.5	3.0	13.3	35.6	28.8	N/A	[53]
*Ascophylum nodosum*	93.2	29.5	11.4	3.0	N/A	34.5	18.9	12.9	[11]
*Sacharina latissimi*	94.0	39.9	15.2	1.5	N/A	21.7	8.0	2.8	[11]
*Chlorophyceae* (green)									
*Ulva* sp.	93.6	51.3	14.6	1.15	N/A	21.0	7.45	3.2	[11]
*Rhodophyceae* (red)									
*Halymenia palmata*	90.6	19.0	18.5	1.69	1.83	N/A	N/A	N/A	[54]
*Palmaria palmata*	93.6	21.0	26.8	8.0	N/A	N/A	N/A	N/A	[55]

**Table 2:** Main effects of dietary seaweed products on pork quality.

Seaweed	Incorporation Rate/Type of Product	Animal and Initial Live Weight	Main Findings	Reference
*Macrocystis pyrifera* (brown)	0%, 2%, and 4% dietary seaweed meal	Castrated male and female pigs 52.5 ± 2.8 kg	Meat from pigs fed with 4% seaweed had less red intensity (a*) compared to control and 2% group Meat ash content was increased in 4% group compared to the other groups, albeit having significantly less Mn, Fe, and Cu	[59]
*Ascophylum nodosum* (brown)	0% and 2% dietary seaweed meal	Male and female piglets (Seghers hybrid × Pietrain) 6.88 ± 1.21 kg	Significant accumulation of iodine in several tissues, including muscle	[60]
*Laminaria digitata* (brown)	500 and 420 mg/kg of feed of laminarin and fucoidan, respectively	Male and female pigs (Large White × Landrace) 14.51 kg	Decreased lipid oxidation (TBARS) in the muscle of supplemented pigs	[61]
*Laminaria digitata* (brown)	450 and 900 mg/kg of feed of laminarin and fucoidan	Male and female pigs (Large White × Landrace) 82 kg	Lipid oxidation was lower in the *longissimus dorsi* of pigs when supplemented three weeks before slaughter When pigs were supplemented for 6 weeks before slaughter, the total saturated fatty acid content of meat was lower	[62]
*Enteromorpha* sp. (green)	4% (starter), 3% (grower), and 2.5% (finisher) (premix containing enriched biomass with Cu and Zn)	Male and female pigs (Polish Landrace × Polish Large White × Hampshire/Pietrain) 40 kg	No effects on meat or carcass quality parameters	[63]

**Table 3:** Main effects of dietary seaweed products on the quality of poultry meat.

Seaweed	Incorporation Rate/Type of Product	Animal and Initial Live Weight	Main Findings	Reference
*Laminaria japonica* (brown)	0% and 0.5% fermented seaweed meal	Ross broiler chickens	Reduced lipid oxidation of breast/thigh meat mixture	[76]
*Laminaria japonica* (brown)	0%, 0.1%, 0.5%, and 1% of seaweed meal–charcoal (1:1) mixture	Ducks (Cherry berry, SUPER M3 F1)	Cholesterol was significantly reduced in meat of 1% supplemented ducks Supplementation reduced meat lipid oxidation The group of 1% had higher content of C20:5 n-3, C22:6 n-3, and overall n-3 PUFA in meat	[77]
*Ascophylum nodosum* (brown)	0%, 1%, and 2% seaweed meal	Broad Breasted Bronze turkeys 2567 ± 39.1 g	Supplementation increased eviscerated weight Redness of thigh and breast meat was increased in supplemented groups	[78]
*Ascophylum nodosum* (brown)	0%, 0.5%, 1%, and 2% seaweed meal	Broiler chickens	No effect on lipid oxidation Supplementation increased C18:3 n-6 in breast meat and decreased C20:1 n-9 in thigh meat	[79]
*Halymenia palmata* (red)	0%, 0.05%, 0.1%, 0,15%, 0.25% seaweed meal	Ross 308 broiler chickens 45 ± 0.5 g	Linear decrease in cooking loss and drip loss (day 7) with increasing seaweed Linear increase in breast yield and gizzard weight with increasing seaweed	[54]
*Kappaphycus alvarezii* (red)	0%, 0.05%, 0.15%, and 0.5% dried alkaline extract and aqueous extract	Vencobb 400 broiler chickens	No effect on meat or carcass characteristics	[80]
*Ulva lactuca* (green)	0%, 1%, and 3% seaweed meal, replacing maize	Ross chickens	Supplementation increased breast yield and reduced abdominal fat, 3% dietary inclusion increased dressing yield There was no effect on breast meat colour	[81]
